# The Effect of Daily Methylsulfonylmethane (MSM) Consumption on High-Density Lipoprotein Cholesterol in Healthy Overweight and Obese Adults: A Randomized Controlled Trial

**DOI:** 10.3390/nu13103620

**Published:** 2021-10-15

**Authors:** Lindsey Miller, Kari Thompson, Carolina Pavlenco, Vijaya Saradhi Mettu, Hans Haverkamp, Samantha Skaufel, Abdul Basit, Bhagwat Prasad, Julie Larsen

**Affiliations:** 1 Department of Physiology, DeBusk College of Osteopathic Medicine, Lincoln Memorial University, Knoxville, TN 37934, USA; 2 Department of Nutrition and Exercise Physiology, Elson S. Floyd College of Medicine, Washington State University, Spokane, WA 99202, USA; kari.thompson@wsu.edu (K.T.); carolina.pavlenco@wsu.edu (C.P.); hans.haverkamp@wsu.edu (H.H.); samskaufel@gmail.com (S.S.); larsen2@wsu.edu (J.L.); 3 Department of Pharmaceutical Sciences, Washington State University, Spokane, WA 99202, USA; vijaya.mettu@wsu.edu (V.S.M.); abdulbasit.shaikh@wsu.edu (A.B.); bhagwat.prasad@wsu.edu (B.P.)

**Keywords:** methylsulfonylmethane, cardiometabolic, inflammation, overweight, obesity

## Abstract

Interventions to decrease inflammation and improve metabolic function hold promise for the prevention of obesity-related diseases. Methylsulfonylmethane (MSM) is a naturally occurring compound that demonstrates antioxidant and anti-inflammatory effects. Improvements in measures of metabolic health have been observed in mouse models of obesity and diabetes following MSM treatment. However, the effects of MSM on obesity-related diseases in humans have not been investigated. Therefore, the purpose of this investigation was to determine whether MSM supplementation improves cardiometabolic health, and markers of inflammation and oxidative status. A randomized, double-blind, placebo-controlled design was utilized with a total of 22 overweight or obese adults completing the study. Participants received either a placebo (white rice flour) or 3 g MSM daily for 16 weeks. Measurements occurred at baseline and after 4, 8, and 16 weeks. Outcome measures included fasting glucose, insulin, blood lipids, blood pressure, body composition, metabolic rate, and markers of inflammation and oxidative status. The primary finding of this work shows that high-density lipoprotein cholesterol was elevated at 8 and 16 weeks of daily MSM consumption compared to baseline, (*p* = 0.008, *p* = 0.013). Our findings indicate that MSM supplementation may improve the cholesterol profile by resulting in higher levels of high-density lipoprotein cholesterol.

## 1. Introduction

More than seventy percent of the adult population aged 20 and over is classified as overweight or obese in the Unites States [1]. Overweight and obesity are associated with chronic inflammation and oxidative stress, which contributes to metabolic dysfunction and various pathologies including type 2 diabetes, cardiac fibrosis and cardiovascular disease (CVD) [2,3,4,5,6,7,8], referred to collectively as cardiometabolic disease. Although overweight or obesity alone is a risk factor for cardiometabolic diseases, the presence of metabolic dysfunction, known as metabolically unhealthy overweight/obesity, further increases risk for the development of CVD and type 2 diabetes compared to individuals with metabolically healthy overweight/obesity [9,10,11,12,13,14]. Although the precise definition of ‘metabolically unhealthy’ has varied in the literature, the most common definition is based upon the presence of metabolic syndrome [9]. Metabolic syndrome is defined by the International Diabetes Federation as the presence of three or more of the following risk factors: central obesity measured by elevated waist circumference based upon population and country specific definitions, raised triglycerides (≥150 mg/dL), reduced high-density lipoprotein (HDL) cholesterol (<40 mg/dL in males and <50 mg/dL in females) or receiving medications for previously diagnosed dyslipidemia, raised blood pressure (systolic ≥ 130 mmHg or diastolic ≥ 85 mmHg) or treatment for previously diagnosed hypertension, and raised fasting plasma glucose ≥100 mg/dL) or previously diagnosed type 2 diabetes [15]. Weight loss programs are often considered the first intervention to improve health outcomes in overweight or obese individuals; however, many programs are unsuccessful, or weight loss is unable to be maintained [16]. Therefore, additional strategies to improve the inflammatory status and metabolic profile, i.e., a shift from metabolically unhealthy to metabolically healthy overweight/obesity, are of the utmost importance for reducing disease development and mortality in obese individuals [13,17].

Recent reports have demonstrated methylsulfonylmethane (MSM), a common dietary supplement, has anti-inflammatory effects both in vitro and in vivo [18,19,20]. We have recently found that MSM decreases responsiveness to pro-inflammatory stimuli in a model of human cardiac cells, specifically by decreasing the transcript and protein expression of interleukin-6 (IL-6) [21]. Chronic elevation of IL-6 is associated with decreased insulin sensitivity and the development of type 2 diabetes and cardiovascular disease [2,6,22]. Furthermore, MSM improves glucose tolerance and insulin sensitivity in mouse models of obesity and type 2 diabetes [23]. Overall, evidence supports that MSM would have a beneficial effect on obesity-related diseases. However, the effects of MSM on cardiometabolic health and disease risk profiles in humans remained unknown. Therefore, the aim of this study was to test the hypothesis that MSM consumption would positively impact markers of metabolic health, inflammation, oxidative status, and cardiac fibrosis in individuals with overweight or obesity.

## 2. Materials and Methods

### 2.1. Study Approval

The study protocol was registered on ClinicalTrial.org (#NCT03716791) and approved by the Washington State University Institutional Review Board, #16970—009.

### 2.2. Participant Recruitment

A randomized, placebo-controlled, double-blind study design was utilized. Participants were recruited by advertisements on social media, flyers posted in public spaces, and campus announcements. Once potential participants contacted the study team, additional information and a pre-screen survey was provided to assess whether they met the study inclusion criteria. Inclusion criteria assessed with the pre-screen survey included being 18–65 years of age; having a BMI of 25 kg/m^2^ or above; free from cardiovascular, liver, renal, thyroid, muscular disease, diabetes, and cancer; not taking immunosuppressants or medication to decrease blood pressure, cholesterol, or blood sugar; not currently taking and willing to refrain from taking dietary supplements; not habitually smoking; not currently pregnant, breastfeeding, or planning to become pregnant during study duration; and willing to maintain regular diet and physical activity levels during the study. After completing the pre-screen survey and signing the informed consent, measured exclusion criteria included a BMI less than 25 kg/m^2^, fasting blood glucose greater than 125 mg/dL, systolic blood pressure 180 mmHg or greater, or diastolic blood pressure 120 mmHg or greater.

**Figure 1:**
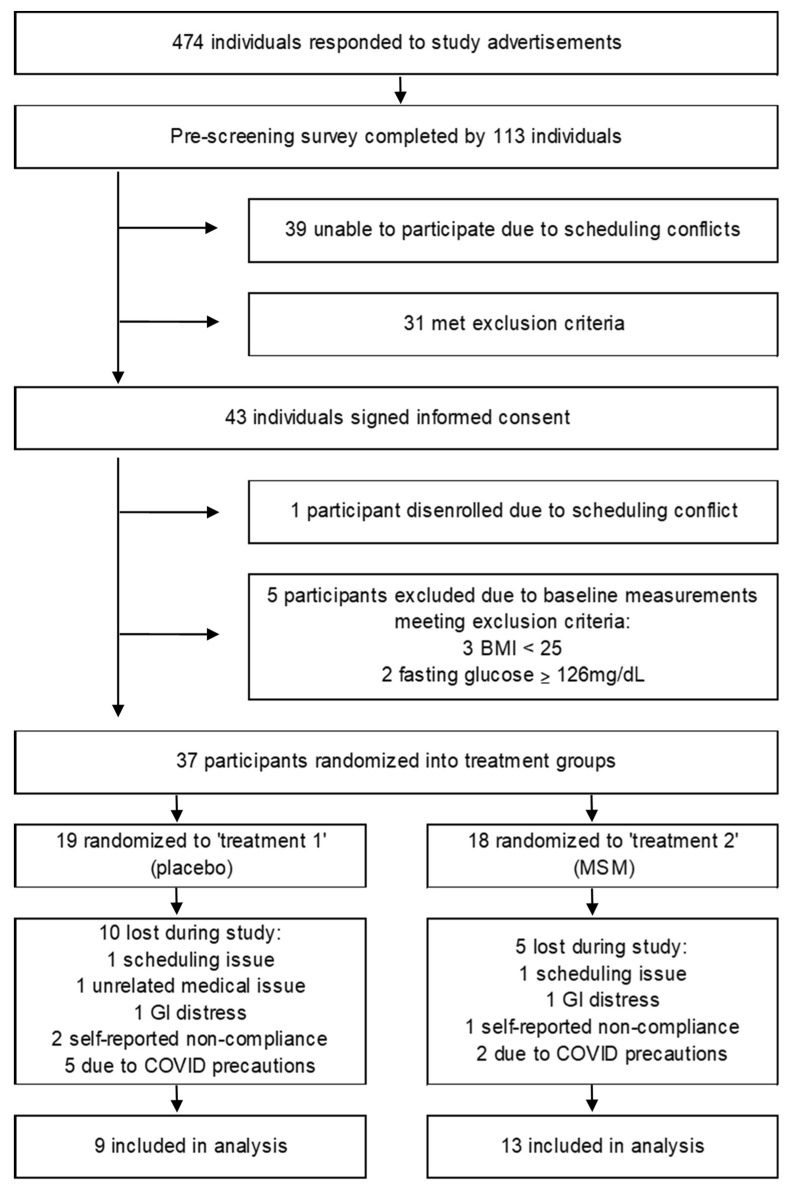
Flow diagram of participant recruitment process. BMI, body mass index; MSM, methylsulfonylmethane.

The required sample size was estimated by performing a power analysis based upon effect sizes of our key selected outcomes from previously published work. At a power of 0.800 and alpha 0.0500, we predicted we would need a sample size of 10 participants completing the study, per group. With an estimated rate of attrition of 30%, we aimed to recruit 14 participants per group. In the original study design, we aimed to have 10 men and 10 women complete the study for each group to allow for analysis of potential differences between sexes. Unfortunately, the target of men and women participants was not met due precautions taken in response to SARS-CoV-2, and thus we examined two groups with a mix of men and women. A flow diagram of the participant recruitment process is shown in Figure 1. A total of 474 individuals responded to the advertisements. The pre-screening survey was completed by 113 individuals, 31 of which were excluded due to not meeting the inclusion criteria and 39 of which were unable to participate due to scheduling challenges. The remaining 43 individuals signed the informed consent, with 1 individual later disenrolling from the study prior to baseline measurements due to scheduling issues. A total of 42 adults completed baseline measurements, with 22 completing the entire study. Of the 20 individuals who were unable to complete the study, 5 were excluded due to baseline measure exclusion criteria, including 3 with measured BMI being below the study inclusion criteria, and 2 participants excluded due to fasting blood glucose measures above study inclusion criteria. Three participants were excluded due to self-reported non-compliance (for example eating prior to visits, regularly forgetting to consume supplement), 2 for unforeseen scheduling issues, 1 medical/personal issue unrelated to the study, 2 reported gastrointestinal disturbance (1 from each treatment group), and 7 due to COVID-related social distancing requirements. 

### 2.3. Study Design

Participants were randomized to receive 3 g/day of MSM (OptiMSM^®^, Bergstrom Nutrition, Vancouver, WA, USA) or placebo (capsules containing white rice flour) daily for 16 weeks (blinded from investigators and participants as ‘treatment 1’ or ‘treatment 2’). Both MSM and placebo consisted of 3 pills taken together with or without food in the morning. Outcomes were measured at baseline (pre-supplementation) and following supplementation at 4, 8, and 16 weeks. Participants completed physical activity questionnaires and 3-day diet recalls at baseline and week 16 to ensure no significant changes in diet and physical activity occurred over the course of the study. At each visit, participants were provided enough treatment to last to the next scheduled visit and were instructed to return any unused pills at the next visit for adherence determination. Baseline participant characteristics for each group are presented in Table 1. Prior to the visit, participants were instructed to avoid eating or drinking anything other than water for at least 12 h, including the supplement; avoid over-the-counter medications for 24 h; avoid consuming alcohol or using nicotine products for 24 h; and to avoid vigorous exercise for 24 h. Compliance with pre-visit instructions was assessed by verbal questionnaires at each visit. Participants were also asked to report any side-effects experienced and whether they believed they were consuming MSM or placebo at each follow-up visit. 

### 2.4. Physical Activity

Participants agreed to avoid any changes in their physical activity habits for the duration of the study. Participants completed the physical activity questionnaire developed by Rubenstein, Morgenstern, and colleagues (PAQ-M) [24]. The PAQ-M includes eight activity domains enabling the calculation of a total physical activity score in kcal/kg/week and has been validated against the Paffenbarger PAQ [24]. The PAQ-M was completed during the initial visit and again during the last visit to assess potential changes in physical activity that might have occurred through the duration of the study. 

### 2.5. Dietary Intake

Participants agreed to avoid any changes in their typical dietary habits for the duration of the study. Participants were asked to complete three separate 24-h diet recalls (2 weekdays and one weekend day) within one week of study start date and again within one week of study end date using the Automated Self-Administered 24-h Dietary Assessment Tool (ASA24) [25]. Each recall was completed within 24 h of the reported day.

### 2.6. Anthropometric Measures

Height was measured at each visit using a stadiometer. Body weight, body mass index (BMI), and percent body fat assessed by bioelectric impedance were measured at each visit with the Tanita body composition analyzer SC-240. Waist and hip circumference were also measured at each visit. 

### 2.7. Metabolic Rate and Blood Pressure

Resting heart rate (bpm) and blood pressure (mmHg) were measured at each visit using an OMRON HEM-907XL digital blood pressure monitor, following 5 min of seated rest. Resting metabolic rate (kcal/day) was assessed at baseline and at the 16-week visit utilizing either a ParvoMedics TrueOne 2400 or a Carefusion Vyntus CPX, with each participant completing both of their measurements on the same system. Metabolic measurements were recorded for 15 min of seated rest, with the last 5 min of data averaged for determination of metabolic rate and respiratory exchange ratio (RER). 

### 2.8. Blood Measures

Blood was collected from the antecubital vein by a registered nurse. Blood collected in serum separator tubes was sent to Laboratory Corporation of America (LabCorp) for analysis of glucose, insulin, total cholesterol, high-density lipoprotein cholesterol levels (HDL), low-density lipoprotein levels (LDL), very low-density lipoprotein (VLDL), triglyceride levels, and C-reactive protein (CRP). Insulin sensitivity was assessed by the Homeostatic model assessment for insulin resistance (HOMA-IR), calculated as the product of fasting glucose (mmol/L) and fasting insulin (uIU/mL) divided by 22.5 [26,27]. For all other measures, blood was collected in serum separator tubes, allowed to clot at room temperature for 15–20 min, and centrifuged at 4000× *g* for 15 min. Serum was aliquoted and stored at −80 °C. Additional markers of oxidative status and inflammation were assessed using commercially available kits, following manufacturer’s instructions. Assay manufacturer information is as follows: interleukin-6 (IL-6) Invitrogen, Vienna Austria, Cat# KHC0061; tumor necrosis factor-α (TNF-α) Invitrogen, Vienna Austria, Cat# KHC3014; total antioxidant capacity (TAC) Cayman Chemical, Ann Arbor, MI, USA, Item# 709001; Trans-4-Hydroxy-2-nonenal (4-HNE), Novus Biologicals, Littleton, CO, USA, Cat#NBP2-66364. Procollagen I N-terminal peptide (PINP) and procollagen III N-terminal peptide (PIIINP) were measured to assess cardiac fibrosis, purchased from Cloud-Clone Corp., Wuhan, China Cat# SEA957Hu and Cat# SEA573Hu, respectively. Serum concentrations of MSM were determined by LC-MS/MS following a previously developed protocol [28]. Briefly, 20 µL of human serum samples were precipitated with 50 µL of acetone containing 112 µg/mL of internal standard (methyl-D3 methanesulfonate). Samples were vortexed and acetone was removed by evaporation on heating blocks at 37 °C for 1 h. The samples were diluted by adding 150 µL of water with 0.1% formic acid and vortex mixed for 5 min before centrifugation at 16,000× *g* for 20 min. The supernatant was injected into LC-MS/MS. LC-MS/MS analysis was carried out using an M-class Waters UPLC system coupled with Xevo TQ-XS µLC-MS/MS instrument (Waters Technologies, Milford, MA, USA). Chromatographic separation of MSM and its internal standard, methyl-D3 methanesulfonate, was achieved using a reversed phase BEH C18 column (130 Å, 1.7 µm, 1 mm × 100 mm, Waters Technologies). Mobile phase consisted of eluent A (1 mM ammonium acetate with 0.1% formic acid in water) and B (methanol with 0.1% formic acid). The following gradient was applied to separate the analyte and internal standard: 0.0–1 min (B 10% to 50%), 1–4 min (B % to 95%), 4–5 min (B 95%), and 5.1–6 min (B 10%). The flow rate was 25 µL/min from 0 to 1 min and increased to 35 µL/min from 1 to 5 min, and reduced to 25 µL/min until 6 min. The mass spectrometer was used in the positive ESI mode using the multiple reaction monitoring approach. The capillary voltage was set to 3.5 kV, source temperature 150 °C, desolvation temperature was 250 °C, the gas flow to cone was 150 L/h and to the desolvation was 600 L/h. The multiple reaction monitoring transitions were *m*/*z* 94.9 > *m*/*z* 62.9 and *m*/*z* 113.54 > *m*/*z* 78.94 for MSM and methyl-D3 methanesulfonate, respectively. Data were acquired by TargetLynx software (MassLynx version 4.2 (Targetlynx, Waters Technologies, Milford, MA, USA)).

### 2.9. Statistics

Significance was indicated by *p* values of ≤0.05, a priori. The Shapiro–Wilk test was utilized to confirm normality and differences between groups and across timepoints were assessed by two-way repeated measures ANOVA with Tukey post-hoc analysis when appropriate. In the case of failed normality, rank transformation was performed. *t*-tests were used to examine differences between groups at baseline with Mann–Whitney Rank Sum Test in the case of failed normality. Within the group receiving MSM, Pearson r was used to evaluate correlations between outcome measures and serum MSM concentrations. SigmaPlot software was utilized for all statistical analyses.

## 3. Results

### 3.1. Physical Activity and Diet

No group differences were detected at baseline for physical activity (kcal/kg/week), total daily caloric intake, or grams of carbohydrate, or protein, although fat intake was higher in the group receiving MSM (Table 1). Additionally, no differences were noted between the baseline (0 week) and end of study (16 week) for any variable in either group, (data not shown).

### 3.2. Serum Methylsulfonylmethane

**Figure 2:**
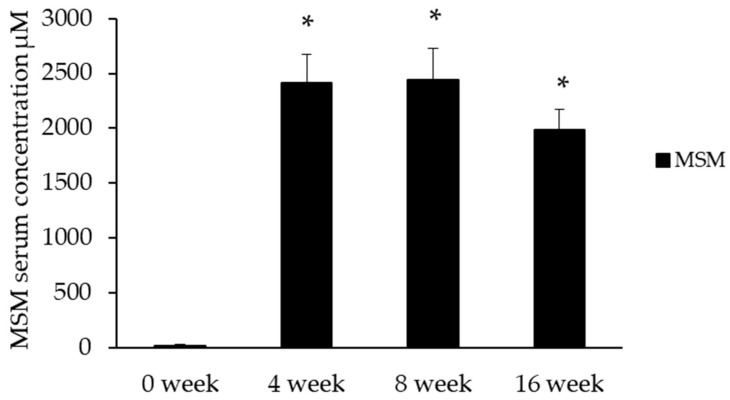
Serum MSM concentrations were significantly elevated after 4 weeks of supplementation in the MSM group. MSM was undetectable in serum samples from placebo at each time point. * Indicates significantly different from baseline, *p* ≤ 0.001.

Serum MSM concentrations were significantly elevated by 4 weeks in the MSM group (Figure 2).

### 3.3. Anthropometric, Metabolic Measures

**Figure 3:**
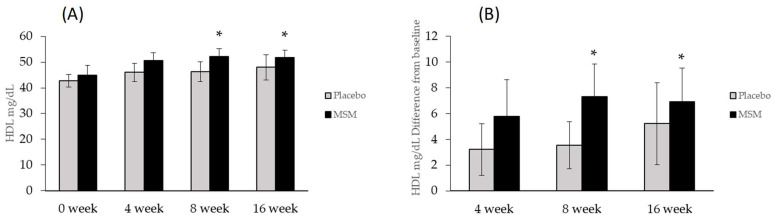
High-density lipoprotein cholesterol (HDL) was elevated at 8 and 16 weeks in the MSM group. (**A**) absolute values of HDL, (**B**) Differences from baseline. * Indicates different from baseline, *p* ≤ 0.05.

Anthropometric measures consisted of height, weight, body mass index (BMI), waist circumference, waist–hip circumference ratio, body composition by bioelectric impedance, resting metabolic rate, resting heart rate, and blood pressure. No differences were detected between groups at baseline for any measure. Additionally, no differences were found between groups for any of the anthropometric measures, blood pressure, resting metabolic rate, or respiratory exchange ratio during treatment. Additionally, no differences were found at baseline, or any time point for fasting blood glucose, insulin, HOMA index, total cholesterol, LDL, VLDL, or triglycerides (Table 2). However, in the experimental group HDL was increased at 8 and 16 weeks compared with baseline (*p* = 0.008, *p* = 0.013), although there were no significant differences between groups (Figure 3).

### 3.4. Measures of Inflammation, Oxidative Status, and Cardiac Fibrosis

**Figure 4:**
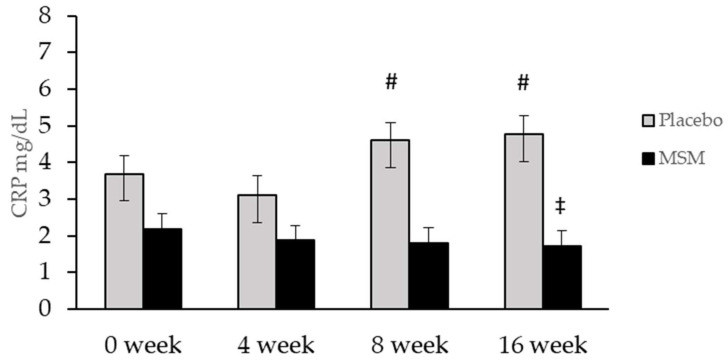
C-reactive protein (CRP) levels were lower in the MSM group compared to placebo at week 16. However, the placebo group showed increased CRP levels at week 8 and 16 compared to week 4. # Significantly different from week 4, *p* ≤ 0.05; ‡ significantly different from placebo group, *p* ≤ 0.05.

No differences were detected for TNFα or IL-6, (Table 3). CRP was significantly lower in MSM compared to placebo at week 16 (*p* = 0.04). However, CRP increased across time in the placebo group, with significant differences found from week 4 compared to week 8 and 16 (*p* = 0.039, *p* = 0.021) while CRP was unchanged in the MSM group (Figure 4). The total antioxidant capacity of the serum was not different between the MSM and placebo group (Table 3). No differences were found between the groups for PIIINP or PINP, (Table 3). No significant correlations were found between serum MSM concentration and any outcome measure.

## 4. Discussion

The purpose of this study was to determine the effect of MSM on markers of cardiometabolic health. Our major finding is that daily MSM consumption resulted in an improvement in HDL levels at 16 weeks compared to baseline. Additionally, CRP levels were lower in the group receiving MSM compared to the placebo group at 16 weeks, although this was due to increased CRP levels in the placebo group. Strengths of the study include utilization of a randomized double-blind placebo-controlled design and quantification of MSM levels in serum. However, the small sample size limits interpretation. More studies utilizing larger participant numbers and a cross-over study design are warranted to further characterize the effects of MSM on inflammation, oxidative stress, and cardiac fibrosis in humans. Our findings in the context of previous work and suggestions for future studies are discussed below.

### 4.1. Physical Activity and Diet

Our data show that typical physical activity or dietary habits were not altered during the study period. Groups did not differ on levels of physical activity or caloric intake, although fat intake was greater in the group receiving MSM, which we are unable to account for. Overall, relatively low levels of physical activity were reported by the participants. While beyond the scope of the current study, future studies should seek to identify whether physical activity level or dietary factors may affect MSM activity.

### 4.2. Serum MSM

Serum MSM concentrations were significantly elevated by 4 weeks of MSM supplementation and did not differ between weeks 4 and 16. However, we observed a non-significant decrease in serum MSM concentration at week 16, which we are unable to explain from the current data set. The concentration of serum MSM found in participants consuming 3 g of MSM daily is similar to that found in a previous study investigating the serum concentrations following varying doses of MSM consumption [29]. Additional studies examining more prolonged intake of MSM and serum concentrations would shed light on whether prolonged, regular supplementation results in steady serum concentrations, or if serum levels may change over time.

### 4.3. Markers of Cardiometabolic Health

To our knowledge, this is the first investigation of the potential benefits of MSM supplementation on cardiometabolic health in humans. Rationale for the study design stemmed from established links between cardiometabolic disease and inflammation and previous research indicating an anti-inflammatory effect of MSM [18,19,20,23]. Importantly, MSM has been found to improve insulin sensitivity and blood glucose levels in mouse models of diet-induced obesity as well as leptin receptor-deficient (db/db) mice [23]. Although the mechanisms of MSM action to improve metabolic function are yet to be fully resolved, it appears modulation of gene expression impacting lipogenesis and inflammation is involved [23]. MSM improved glucose levels in db/db mice in a dose-dependent fashion with corresponding increases in insulin level and sensitivity, with the highest dose completely normalizing glucose levels [23]. Interestingly, MSM supplementation in normoglycemic mice does not alter blood glucose levels [23]. 

Our results do not indicate that 16 weeks of MSM supplementation causes change in fasting glucose levels or insulin sensitivity. However, when interpreting the results, it is important to note that the range in fasting glucose and markers of insulin sensitivity at baseline among participants was in the ‘normal’ range (some participants considered normal, while others ‘high’), and the low statistical power due to small sample size. Since MSM has not been found to impact glucose levels or insulin sensitivity when values are in the normal range, it may be that individuals without impaired glucose levels or insulin sensitivity would not experience changes in these values with MSM supplementation. Therefore, further studies with a larger sample size and more homogenous participant characteristics of metabolically unhealthy obesity need to be performed.

A primary, novel finding of the present study is the improvement in HDL cholesterol levels in participants consuming MSM. Epidemiological studies have reported the inverse relationship between HDL and cardiovascular disease, with an estimated decrease in coronary heart disease risk of 2% in men and 3% in women for every 1 mg/dL increase in HDL cholesterol [30]. Therefore, MSM supplementation could offer significant health benefits through elevated HDL levels. However, it is important to note that some studies of pharmacologically elevated HDL cholesterol have failed to translate to improved health outcomes [31]. Whether MSM impacts the overall function of HDL cholesterol is yet to be determined. Additionally, we are unable to determine the mechanisms for increased HDL cholesterol. A potential link between MSM consumption and improved HDL cholesterol levels could be due to decreased inflammation and changes in the metabolism of HDL. Although our findings did not support decreased levels of inflammatory markers, multiple studies have indicated the anti-inflammatory nature of MSM [19,20,32]. A specific mechanism could be through the effect of MSM on NF-κB activity. Inhibition of NF-κB results in elevated HDL cholesterol [33], and MSM has been documented to decrease NF-κB activation in LPS stimulated macrophages [19], thus presenting a plausible mechanism for our present observations. Future studies to confirm our observations and further investigate the effect of MSM on HDL function are warranted. 

### 4.4. Markers of Inflammation and Oxidative Status

To our knowledge, this is the first study to examine the effect of MSM on markers of inflammatory and oxidative status in adults with overweight or obesity. Our study did not find any significant changes in the inflammatory markers TNFα, IL-6, or markers of oxidative status, TAC or 4-HNE. C-reactive protein was significantly higher in the placebo group than the treatment group at 16 weeks, although this was due to an increased CRP level in that group, without any change in CRP levels in the group receiving MSM. Although preventing a rise in CRP levels may be considered a benefit [34], we are unable to account for the cause of increased CRP level in the placebo group. Therefore, the data are inconclusive on whether 16 weeks of MSM supplementation resulted in improvement in inflammatory profile based upon our current data set. 

In contrast to our findings, recent evidence suggests that MSM may decrease levels of inflammation and oxidative stress [18,20,32,35,36]. For example, MSM inhibits nod-like receptor family pyrin domains containing 3 (NLRP3) inflammasome in macrophages derived from humans and mice, treated with lipopolysaccharide [20]. Transcript expression of IL-1α, IL-1β, IL-6, and NLRP3 were decreased following MSM treatment [20]. Several studies have demonstrated decreased measures of inflammation and oxidative stress following MSM supplementation in models of exhaustive exercise, although pre-exercise measures do not appear to be impacted [18,32,35,36]. It may be that overall levels of the measured inflammatory markers in the present study were too low to detect changes in ‘unstressed’ situations, similar to studies that have examined the effect of MSM at rest and during exhaustive exercise. 

### 4.5. Cardiac Fibrosis

Interventions to decrease NF-κB expression, inflammatory cytokines, and ROS decrease cardiac fibrosis [37,38]. Since previous studies indicated MSM decreases NF-κB activation and downstream inflammatory markers, we hypothesized that MSM could also decrease markers of cardiac fibrosis. Markers of fibrosis were measured at baseline and 16 weeks by examining blood levels of procollagen III N-terminal peptide (PIIINP) and procollagen I N-terminal peptide (PINP). These markers decrease and increase, respectively, in accordance with the development of cardiac fibrosis [39]. Our data do not support this hypothesis, since PIIINP and PINP were not different between groups. Study limitations (sample size, relatively healthy population) and lack of more sensitive measures of cardiac fibrosis should be noted. More studies are needed to investigate the potential effects of MSM on cardiac fibrosis. 

## 5. Conclusions

In conclusion, oral supplementation with MSM may offer cardiometabolic benefits through increased HDL cholesterol. Animal models suggest that MSM may also improve insulin sensitivity and glucose regulation. The lack of supportive evidence in the present study should be interpreted with caution due to lack of a cross-over study design and homogeneity among our participant population (i.e., normal vs. high fasting blood glucose levels) and the relatively small sample size. Future work is needed to determine the full benefits of MSM on cardiometabolic health.

## Figures and Tables

**Table 1:** Baseline Characteristics.

Group	Placebo	MSM	*p* Value
*n* (sex)	6 (female) 3 (male)	9 (female) 4 (male)	
age	40.7 ± 4.0	43.7 ± 3.9	0.605
BMI (kg/m^2^)	34 ± 2.3	31.5 ± 1.1	0.593
% body fat	41.2 ± 3.3	39.8 ± 2.0	0.708
RMR (kcals/day)	1650.0 ± 218.3	1596.9 ± 109.3	0.814
PA (kcals/kg/week)	290.5 ± 29.2	299.6 ± 24.3	0.789
Dietary intake (kcals/day)	2069.1 ± 174.4	2206.0 ± 134.3	0.54
Fat (g/day)	78.9 ± 6.5	102.2 ± 6.5	0.031 *
Protein (g/day)	82.6 ± 11.0	97.8 ± 10.2	0.263
Carbohydrate (g/day)	258.0 ± 43.5	231.5 ± 25.4	0.583

BMI, body mass index; RMR, resting metabolic rate; PA, physical activity. * indicates a significant difference between groups, *p* ≤ 0.05.

**Table 2:** Measures of cardiometabolic health. HOMA-IR, Homeostatic model assessment for insulin resistance; TGs, triglycerides; LDL, low-density lipoprotein; VLDL, very-low-density lipoprotein; HDL, high-density lipoprotein; SBP, systolic blood pressure; DBP, diastolic blood pressure. * indicates different from baseline, *p* ≤ 0.05.

Variable	Group	Baseline	4 Week	8 Week	16 Week	*p* Value
glucose (mg/dL)	placebo	92.3 ± 4.1	93.8 ± 3.7	92.9 ± 3.9	94.4 ± 3.2	0.656
	MSM	92.5 ± 3.0	95.0 ± 2.8	93.8 ± 2.9	93.2 ± 2.5	
insulin (uUI/mL)	placebo	19.6 ± 3.6	18.2 ± 4.4	17.4 ± 5.4	20.6 ± 5.8	0.097
	MSM	18.5 ± 2.6	18.3 ± 1.7	14.7 ± 1.5	17.9 ± 1.6	
HOMA-IR	placebo	4.5 ± 0.8	4.2 ± 1.1	4.0 ± 1.3	4.8 ± 1.4	0.260
	MSM	4.2 ± 0.6	4.3 ± 0.4	3.9 ± 0.5	4.1 ± 0.4	
Total Cholesterol (mg/dL)	placebo	188.3 ± 11.5	194.3 ± 8.0	200 ± 9.4	186.9 ± 9.3	0.099
	MSM	172.2 ± 10.3	179.0 ± 8.4	181.2 ± 8.0	176.4 ± 8.3	
TGs (mg/dL)	placebo	165.0 ± 36.0	121.1 ± 13.8	156.4 ± 35.7	162.1 ± 26.2	0.515
	MSM	112.5 ± 11.4	110.7 ± 9.6	112.1 ± 12.8	131.6 ± 18.3	
LDL (mg/dL)	placebo	118.6 ± 9.2	123.9 ± 6.5	127.6 ± 9.7	109.7 ± 10.2	0.126
	MSM	102.0 ± 8.5	106.2 ± 7.3	107.4 ± 5.6	100.2 ± 6.3	
VLDL (mg/dL)	placebo	27.8 ± 3.4	24.4 ± 2.7	27.1 ± 1.4	29.2 ± 5.2	0.235
	MSM	22.5 ± 2.3	22.2 ± 1.9	21.6 ± 2.6	24.4 ± 3.6	
HDL (mg/dL)	placebo	42.7 ± 2.5	46.0 ± 3.8	46.3 ± 3.8	48.0 ± 4.9	0.003
	MSM	44.9 ± 3.7	50.7 ± 3.0	* 52.2 ± 2.9	* 51.8 ± 2.8	
SBP (mmHg)	placebo	123.5 ± 5.2	118.1 ± 5.2	119.3 ± 5.4	114.9 ± 3.1	0.625
	MSM	127.3 ± 4.6	120.3 ± 5.2	119.7 ± 4.0	121.6 ± 4.8	
DBP (mmHg)	placebo	84.8 ± 4.5	77.6 ± 3.5	79.8 ± 3.0	78.2 ± 2.6	0.967
	MSM	80.7 ± 3.1	81.3 ± 4.0	77.6 ± 3.5	80.1 ± 3.3	

**Table 3:** Markers of inflammation, oxidative status, and cardiac fibrosis. All measures expressed relative to total serum protein content. TAC, Total antioxidant capacity; TNF-α, Tumor necrosis factor-α, IL-6, Interleukin-6; 4-HNE, Trans-4-Hydroxy-2-nonenal; PINP, Procollagen I N-terminal peptide; PIIINP, procollagen III N-terminal peptide.

Variable	Group	Baseline	4 Week	8 Week	16 Week	*p* Value
TAC	placebo	6.06 ± 0.58	6.49 ± 0.78	6.30 ± 0.74	6.44 ± 0.91	0.583
	MSM	6.37 ± 0.58	6.49 ± 0.61	6.75 ± 0.66	6.75 ± 0.60	
TNF-α	placebo	6.46 ± 1.08	6.63 ± 1.07	6.72 ± 1.07	6.84 ± 1.08	0.498
	MSM	7.84 ± 1.06	7.76 ± 1.00	7.63 ± 0.98	8.04 ± 1.13	
IL-6	placebo	1.75 ± 0.43	2.33 ± 0.59	2.17 ± 0.71	2.29 ± 0.66	0.319
	MSM	1.79 ± 0.60	2.07 ± 0.67	1.76 ± 0.62	1.68 ± 0.57	
4-HNE	placebo	91.24 ± 20.91	96.63 ± 22.14	87.77 ± 21.5	85.75 ± 26.62	0.574
	MSM	50.90 ± 6.58	55.01 ± 7.37	55.61 ± 8.51	49.88 ± 7.54	
PINP	placebo	437.38 ± 98.32	_	_	363.27 ± 51.39	0.297
	MSM	432.74 ± 77.78	_	_	460.38 ± 64.39	
PIIINP	placebo	0.446 ± 0.130	_	_	0.680 ± 0.293	0.308
	MSM	0.305 ± 0.077	_	_	0.533 ± 0.120	
PINP/PIIINP	placebo	3691.512 ± 2714.35	_	_	2369.616 ± 945.108	0.311
	MSM	1727.916 ± 601.482	_	_	899.000 ± 187.634	
